# Fish Skin and Gill Mucus: A Source of Metabolites for Non-Invasive Health Monitoring and Research

**DOI:** 10.3390/metabo12010028

**Published:** 2021-12-31

**Authors:** Lada Ivanova, Oscar D. Rangel-Huerta, Haitham Tartor, Mona C. Gjessing, Maria K. Dahle, Silvio Uhlig

**Affiliations:** Norwegian Veterinary Institute, P.O. Box 64, N-1431 Ås, Norway; oscar.daniel.rangel.huerta@vetinst.no (O.D.R.-H.); haitham.tartor@vetinst.no (H.T.); mona.gjessing@vetinst.no (M.C.G.); maria.dahle@vetinst.no (M.K.D.); silvio.uhlig@vetinst.no (S.U.)

**Keywords:** gill mucus, skin mucus, Atlantic salmon, biomarkers, non-invasive sampling, data normalization

## Abstract

Mucous membranes such as the gill and skin mucosa in fish protect them against a multitude of environmental factors. At the same time, changes in the molecular composition of mucus may provide valuable information about the interaction of the fish with their environment, as well as their health and welfare. In this study, the metabolite profiles of the plasma, skin and gill mucus of freshwater Atlantic salmon (*Salmo salar*) were compared using liquid chromatography coupled to high-resolution mass spectrometry (LC-HRMS). Several normalization procedures aimed to reduce unwanted variation in the untargeted data were tested. In addition, the basal metabolism of skin and gills, and the impact of the anesthetic benzocaine for euthanisation were studied. For targeted metabolomics, the commercial AbsoluteIDQ p400 HR kit was used to evaluate the potential differences in metabolic composition in epidermal mucus as compared to the plasma. The targeted metabolomics data showed a high level of correlation between different types of biological fluids from the same individual, indicating that mucus metabolite composition could be used for fish health monitoring and research.

## 1. Introduction

In order to obtain and maintain high standards in aquaculture, and to reduce mortality, non- or low-invasive methods to monitor fish health are required [1]. In recent years, omics-type applications have accelerated the research in the field of aquaculture. Metabolomics is the holistic study of small molecules, and commonly makes use of advanced analytical chemistry techniques to explore the dynamic metabolic responses of living systems to genetic or environmental factors [1,2]. By using high-resolution mass spectrometry (HRMS) systems coupled with liquid chromatography (LC) or nuclear magnetic resonance spectroscopy (NMR), metabolomics has been successfully used to explore suitable biomarkers for specific conditions, and to identify the particular metabolic pathways that are involved in e.g., disease mechanisms or exposures with certain chemicals [3].

As a result of the increasing focus on animal welfare, the interest in monitoring health biomarkers, e.g., for disease prediction or treatments effects in samples obtained by non-invasive strategies is growing [2]. Blood samples could reflect physiological and/or pathological conditions as it circulates through organs [4,5]. Although blood testing has become one of the most informative screening methods in metabolomics, there is a trend towards using even less invasive samples, like mucus [1]. Considering that the mucus has a wide range of functions including protection, disease resistance, ionic and osmotic regulation and communication, the skin and gill mucus of fish may provide useful and important molecular information on the health state [6]. However, several issues attributed to the sampling of mucus have a strong influence on the validity of metabolomics data [2,7]. Considering the rapid changes in the metabolome, it is crucial to minimize the biological, technical and experimental variability caused by the sampling procedure. Therefore, normalization of the data is highly important to improve reliability and biological relevance of collected data [7]. Taking into account that the mucus layer acts as a barrier between fish and water, an adequate normalization strategy may help to control the variable dilution of mucus samples that greatly affects the concentration of metabolites. In order to overcome this challenge, the comparison of several normalization strategies using untargeted metabolomics data has been shown previously [7,8].

Different mucus components such as proteins, carbohydrates, lipids and other metabolites have been linked to regulatory processes in fish such as osmoregulation, respiration, nutrition or locomotion as well as defense against pathogens [6,9]. Therefore, the idea to use the total metabolic profile of fish mucus to monitor the physiological status of fish and potential responses to environmental disturbances could be promising. However, there are several challenges regarding the suitability of mucus to reflect fish physiology. Recently, it has been demonstrated that gill mucus composition was considerably influenced by several fish life-history traits including the diet, presence of parasites and phylogeny [9]. Epidermal mucus was assumed to better reflect the environmental status of the fish habitat. Several skin mucus metabolites such as free amino acids, glucose, lactate and cortisol were linked to body odor due to the comparable feeding habits and physiological response to stress [10,11]. Furthermore, a recent study reported the positive correlation of stress biomarkers in plasma and skin mucus [12]. Interestingly, the levels of exuded cortisol in mucus were stressor dependent, indicating a physiologically specific response. An earlier study, performed in rainbow trout (*Oncorhynchus mykiss*), reported that amino acid mobilization and metabolism was rapidly affected by elevated plasma cortisol following exhaustive exercise [13]. Thus, altered levels of alanine, glutamine, glutamic acid, and branched-chain amino acids (BCAA), i.e., isoleucine, leucine and valine, were detected in plasma as well as in liver and muscle [13]. For salmonids, a correlation between stress, plasma cortisol levels and several mucus enzymes/proteins has been reported [14]. All this evidence indicates the interrelationship between biological fluids and skin mucus in response to a stimulus. Therefore, one of the goals of the present study was to explore the metabolic profile of skin and gill mucus of salmon by using targeted and untargeted metabolomics. Another aim was to compare metabolite profiles in the mucus with that of plasma samples in order to assess to what degree the mucus metabolome reflects the plasma metabolome. Furthermore, an overdose of benzocaine was used to map the metabolic pathways that could be affected by acute stress associated with the treatment of the fish and hypoxia in order to obtain baseline data for studies that include such (or similar) treatment.

## 2. Results

### 2.1. Untargeted Analyses

#### 2.1.1. Normalization of Data from Skin and Gill Mucus

Considering that the data normalization is a known challenge in metabolomics approaches, several normalization strategies were tested in order to reduce within-class variability attributed to the variable dilution status of individual mucus samples. A total of 20 mucus samples from four biological groups (i.e., *n* = 5 for each of the four groups, gill/skin and control/benzocaine treated) were included in untargeted HILIC–HRMS resulting in the detection of 1605 compounds following pre-processing of the raw data in Compound Discoverer software. In order to find the optimal normalization method to control the impact of variable dilution in the two types of mucus, the dataset was normalized by using the sum of peak areas, sample median, total protein content and median fold change methods. The performance of applied normalization techniques was evaluated by comparing the relative standard deviation (RSD) values of all metabolites within each experimental group. In order to compare the quality of the normalization between different normalization approaches, we calculated the number of metabolites with a RSD ≤ 30% within each group, and the intra-group’s RSD median. Thus, the least successful normalization approach (i.e., resulting in a dataset with high within-group variability) was normalization by total protein content (Table 1). The normalization by using the sample median and median fold change method demonstrated a clear improvement in the data set thereby reducing the variability across sample replicates within the four experimental groups (Table 1).

**Figure 1:**
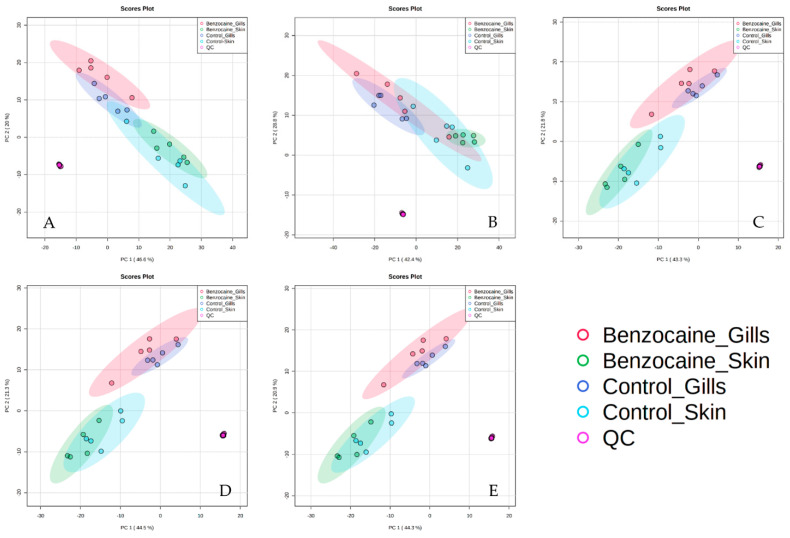
Two-dimensional score plots from PCA of the raw data (**A**) and data normalized by total protein content (**B**), sum (**C**), median (**D**) and median fold change (**E**). The different groups correspond to skin and gill mucus samples collected following percussive stunning (“Control_Skin” and “Control_Gills”) or benzocaine overdosing (“Benzocaine_Skin” and “Benzocaine_Gills”).

For the purpose of direct visual comparison of the normalized data, the four normalization methods in addition to the dataset without normalization were compared in unsupervised principal component analysis (PCA) score plots (Figure 1).

All tested normalization methods resulted in small QC sample offsets (Figure 1). This was also the case for the original data set indicating appropriate quality of the raw data and adequate data filtering in Compound Discoverer software based on 50% QC presence and ≤30% RSD in QCs. When the data was normalized to total protein content (Table S1), the gill and skin mucus samples clustered less clearly (Figure 1). Interestingly, normalization to total protein content slightly deteriorated the clustering between experimental groups, compared to when no normalization was applied. Normalization-by-sum was superior to normalization by protein content. However, the benzocaine and percussive stunning sub-groups were not clearly separated in the PCA score plots and in separate MetaboAnalyst dendrograms generated by using the Euclidean distance (Figure 1 and Figure S1). Normalization-by-median or median fold change resulted in PCA score plots and hierarchical clustering dendrograms that clearly separated the two types of mucus, and also distinguished the samples that were obtained following either benzocaine overdosing or percussive stunning (Figure 1 and Figure S1). The observed trends were confirmed by creating supervised orthogonal partial least squares discriminant analysis (OPLS-DA) models that significantly discriminated between the two types of mucus, as well as between percussive stunning and benzocaine-treated sub-groups (Table 2). The results of permutation tests consisting of 100 permutations confirmed the lack of overfitting. The quality parameters of constructed OPLS-DA models associated with top-performing normalization methods are summarized in Table 2 indicating the reliable discrimination between groups. Considering that comparable levels of the variance (R2Y) and predictability (Q2Y) were demonstrated for both top-performing normalization methods (i.e., normalization-by-median and median fold change), we decided to select the normalization-by-median approach to further explore general metabolic variations between skin and gill mucus and dysregulation followed by anesthesia.

#### 2.1.2. Metabolic Pathways: Functional Analysis

The pathway analysis module in MetaboAnalyst 5.0 (functional analysis, https://www.metaboanalyst.ca, access on 24 December 2021) was used to explore the differences in general metabolism between skin and gill mucus. In addition, we mapped the potential metabolic pathways that could be affected by fish manipulations associated with the mucus harvesting and the use of anesthetic during the sampling procedure. By using an anesthetic (i.e., benzocaine) overdose, we aimed to determine the baseline effect from stress associated with the cessation of breathing which, in turn, reduces gas transfer leading to hypoxia and respiratory acidosis [15].

In general, we found that the major differences in the expression of metabolic pathways, as assessed based on the gill and skin mucus samples, were attributed to the metabolism of free amino acids (Table 3).

An overdose of benzocaine resulted in the disturbance of several metabolic pathways attributed to energy metabolic pathways and amino acid metabolic pathways (Table 3).

It is well known that amino acids regulate key metabolic pathways that are crucial in growth, development and health of fish [16]. Considering that differences in free amino acid metabolism were observed between gill and skin mucus, we decided to use targeted metabolomics based on the AbsoluteIDQ p400 HR kit to evaluate the performance both approaches.

### 2.2. AbsoluteIDQ^®^ p400 HR Kit

#### 2.2.1. Comparing Mucus and Plasma Samples Using Targeted Metabolomics

In order to characterize if the skin and gill mucus metabolome reflected the physiological status of fish, the plasma and mucus samples from the same individuals (*n* = 3) were analyzed by using a targeted approach, and the individual correlations between plasma and mucus metabolites were established.

The final mucus-related dataset, following data filtering described in Section 4.4 included 38 out of the 408 metabolites that are targeted in the AbsoluteIDQ p400 HR kit (Biocrates Life Science AG, Innsbruck, Austria) (Table 4). The median concentrations of all quantified metabolites are presented in the Supplementary Information (Table S2).

**Figure 2:**
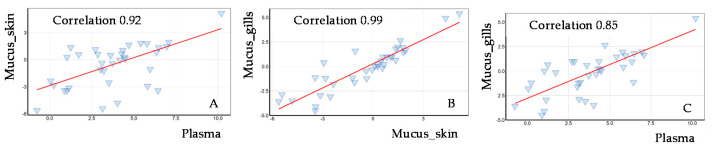
The correlations between concentrations of 36 metabolites that were detected both in plasma and in mucus; (**A**) skin mucus vs. plasma; (**B**) gill mucus vs. skin mucus; (**C**) gill mucus vs. plasma; Pearson’s correlation coefficients were calculated based on normalized concentrations (µM); axes are on a logarithmic scale.

The number of metabolites detected in salmon plasma by using the targeted approach was substantially higher than that in gill or skin mucus. Thus, a total of 163 out of 408 metabolites could be quantified in the salmon plasma samples (Table 4). The median values of quantified plasma metabolites are summarized in Table S3. With exception of SM (42:3) and fumarylcarnitine (AC (4:1-DC)), all metabolites detected in epidermal mucus samples were present in plasma samples. In general, the concentration of metabolites detected in plasma was significantly higher as compared with the skin and gill mucus samples. Clear associations were found between the metabolites that were detected in the three tissues studied. Pearson’s correlation coefficients ranged from 0.85 to 0.99 (Figure 2), indicating that metabolites in epidermal and gill mucus reflect the concentrations in blood, and thus could be used similarly as blood to assess general physiological status of fish.

#### 2.2.2. Skin and Gill Mucus: General Considerations

In general, the overall metabolite concentration in gill mucus was at least twice as compared to the skin mucus samples, which at least in part could be a result of different dilution during the sampling procedure (Table S2). Therefore, the data were additionally normalized by using the median method, which performed best for the untargeted data, in order to allow direct comparison of skin and gill mucus.

Free amino acids were relatively abundant in skin and gill mucus (Table S2 and Figure S2). We detected 19 amino acids in gill mucus, while lysine was not detected in skin mucus, and thus only 18 amino acids were detected in the latter. The amino acid profiles of gill and skin mucus were rather similar, though the gill mucus contained relatively higher amounts of glutamic acid and alanine (Figure S2). In contrast, the skin mucus contained relatively more valine compared to gill mucus. Other metabolites detected in the gill and skin mucus using the AbsoluteIDQ^®^ p400 HR kit included three acylcarnitines and five biogenic amines (Table 4). The group of biogenic amines was largely dominated by taurine that was detected in the 90–500 µM range. Sarcosine was not detected in the skin mucus as compared to the gill mucus. Although it was not expected to find substantial amounts of lipids in skin and gill mucus, we detected eight phosphatidylcholines (PC), one triglyceride (TG) and one sphingomyelin (SM) using the AbsoluteIDQ^®^ p400 HR kit (Table S2).

**Figure 3:**
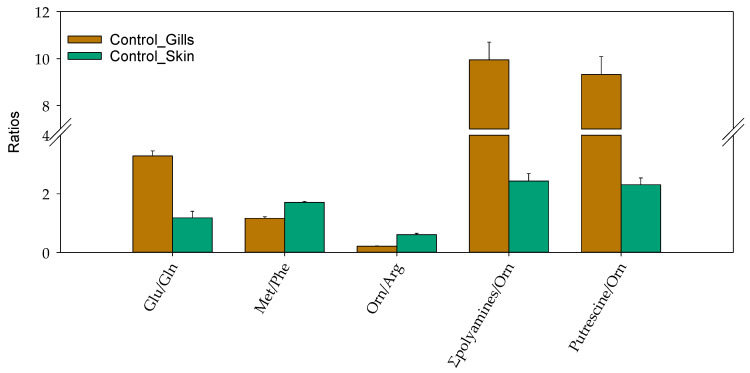
The ratios of metabolites that significantly differentiated the skin and gill mucus samples (Glu, glutamic acid; Gln, glutamine; Met, methionine; Phe, phenylalanine; Orn, ornithine; Arg, arginine; ∑polyamines = (putrescine + spermidine)).

The quantitative data were further used to calculate sums and ratios for several detected metabolites, as such ratios in some cases provide more biological information [17]. Thus, the sum of all glucogenic amino acids and biogenic amines were detected in significantly higher concentrations in gill mucus samples compared to the skin mucus samples (*p*-values of 0.014 and 0.006, respectively; Table S4). In addition, the ratios of glutamic acid to glutamine, sum of putrescine and spermidine to ornithine, as well as putrescine to ornithine demonstrated similar trends (Figure 3). In contrast, the ratios of methionine to phenylalanine and that of ornithine to arginine were significantly lower in gill mucus samples as compared to the skin mucus (Figure 3 and Table S4).

#### 2.2.3. Univariate and Multivariate Analysis: Comparing the Gill and Skin Mucus Metabolome

**Figure 4:**
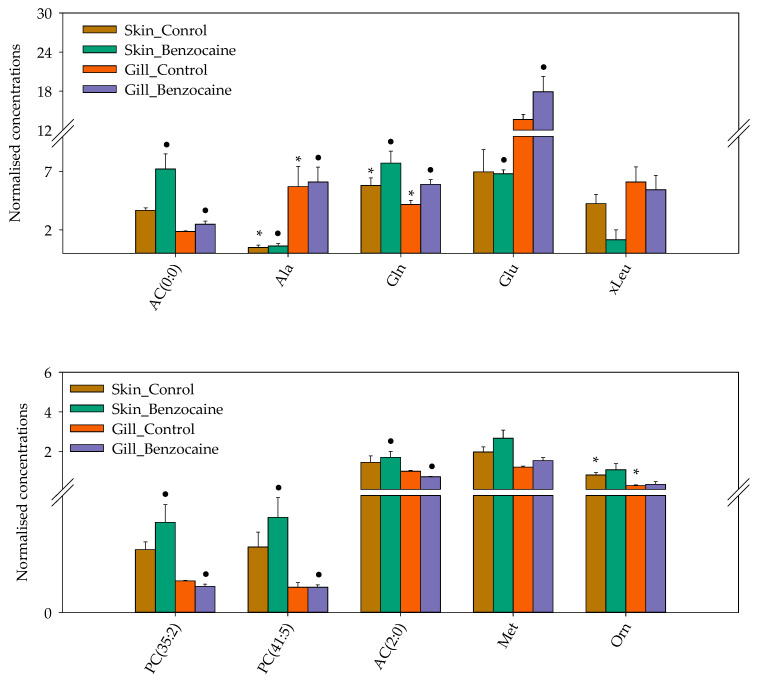
Top 10 most differential metabolites that contributed to differences between gill and skin mucus from salmon euthanized using benzocaine or percussive stunning (“Control”). The metabolite levels (µM) detected with the AbsoluteIDQ^®^ p400 HR kit were normalised by median. The data are presented as the mean, error bars are the standard error. Metabolites that were found in significantly different (FDR-adjusted *p* ≤ 0.1) concentrations with a fold-change ≥2: (*) in skin mucus as compared to the gill mucus and (•) in skin mucus followed by benzocaine treatment. AC (0:0), carnitine; AC (2:0), acetylcarnitine; Ala, alanine; Gln, glutamine; xLeu, sum leucine + isoleucine; Met, methionine; Orn, ornithine.

The pre-processed data were subjected to univariate statistical analyses. Volcano plots showed that three metabolites, i.e., AC (4:1-DC), ornithine and alanine were present in statistically different concentrations in gill and skin mucus (false discovery rate (FDR)-adjusted *p*-value ≤ 0.1 and fold change (FC) ≥ 2) (Table S5). Benzocaine treatment prior to sampling resulted in significantly different concentrations of carnitine (AC (0:0)), alanine, sarcosine, glutamic acid, acetylcarnitine (AC (2:0)), PC (36:5), PC (41:5) and PC (35:2) in skin mucus as compared to the gill mucus (FC = 2; FDR-adjusted *p* = 0.0234; Table S6). A heatmap for visualization of the top 10 most differential metabolites (*t*-test/ANOVA) distinguishing the gill and skin mucus from salmon, euthanized using benzocaine or percussive stunning, can be found in Figure 4 and Figure S3.

**Figure 5:**
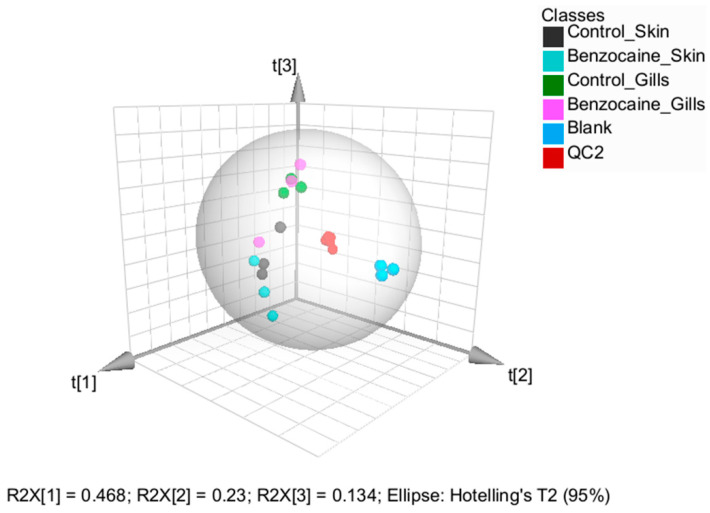
3-D scores plot from unsupervised principal component analysis based on Pareto-scaled and log-transformed data obtained using the AbsoluteIDQ^®^ p400 HR kit. The first three components explain 83% of the total variation. The plot shows that the observations cluster primarily according to the type of tissue mucus was collected from (i.e., skin or gill) and not according to the euthanization method.

Although a PCA score plot revealed a clear separation between gill and skin mucus samples, it did not show obvious variation in the data associated with benzocaine treatments (Figure 5).

Supervised orthogonal partial least squares discriminant analysis (OPLS-DA) to infer metabolites that were discriminant between skin and gill mucus, collected following percussive stunning, did not result in valid models. In addition, OPLS-DA was not able to distinguish between skin and gill mucus collected with or without anesthetics (data not shown). This could be attributed to the limited number of samples used for targeted metabolomics. Although the metabolic profiles of benzocaine treated groups was not clearly distinguishable from control groups, the observed differences between skin and gill mucus were in accordance with the data from untargeted metabolomics, especially with regard to several amino acids, such as alanine, isoleucine and leucine.

## 3. Discussion

The use of skin and gill mucus to monitor and study the health state of fish is of growing interest [1,2,10,11]. Mucus samples may be collected using a non-invasive sampling strategy and provide the opportunity to potentially discover early stages of disease or responses to environmental challenges in general [18]. Although the application and integration of several “omics” techniques could improve the understanding of fish mucus composition, there are several challenges associated with their application in general, especially with metabolomics [1,18]. Special consideration should be given to the sampling procedure, which is a critical source of experimental variability. Several methods could be suitable for the collection of mucus, e.g., gently moving whole fish in plastic bags containing ammonium bicarbonate buffer, scraping of mucus using sterile glass slides, or absorption of mucus using a medical wipe [11,19,20]. Considering that the composition and physical characteristics of both skin and gill mucus can be influenced by several external factors such as dilution with water, the raw data from instrumental analyses must be normalized suitably. Recently, the mucus samples from three model species, meagre (*Argyrosomus regius*, Asso 1801), European sea bass (*Dicentrarchus labrax* L.) and gilthead sea bream (*Sparus aurata* L.) was studied with regard to physiological responses associated with anoxia, infection with *Vibrio anguillarum* and fasting [9]. This study showed that both glucose, lactate and cortisol could serve as mucus biomarkers to monitor physiological responses to environmental or anthropogenic challenges. However, the glucose-to-protein ratio was the most reliable biomarker, indicating the importance of normalization. We therefore compared several normalization strategies to reduce unwanted experimental variability before statistical analyses. Normalization to total protein did not reduce the variability within the experimental groups in our data set. This could in part be explained by the fact that the soluble proteins that were covered by total protein quantification include both proteins that are related to metabolite abundance, such as enzymes, but also transcription factors and structural proteins, which are not related to metabolite abundance directly [7]. In addition, poor protein recovery in mucus, sampled by absorption of the water phase, can be another factor that reduces the suitability of total protein for normalization of metabolite abundance [21].

Although the use of skin mucus as a non-invasive sample for the collection of health-related parameters could be attractive, a clear correlation between the metabolic profile of blood plasma, which is an excellent biofluid for bio-monitoring, and skin mucus has not yet been shown. To our knowledge, only few studies addressed the relation between plasma and mucus metabolites. Such a relation has to some extend been studied for stress biomarkers under different stress conditions [12,22]. Although cortisol was demonstrated to be a more stressor dependent and species-specific biomarker compared to glucose, a strong correlation was demonstrated between the plasma and mucus for both stress biomarkers (r = 0.77) showing the potential of mucus samples to monitor fish welfare [12]. A positive correlation between plasma and mucus cortisol levels in Greater amberjack (*Seriola dumerili*, Risso 1810) was reported by Fernández-Montero et al. (2020) when the fish were exposed to temperature or handling stress, as well as fasting [22]. Although these examples are merely related to stress response and some very few metabolites, our targeted metabolomics data show that mucus metabolite profiles very well reflect those in the plasma. We noticed considerable differences in the abundances of individual metabolites in plasma and mucus, but this did not disrupt the strong correlation between metabolite patterns in these biofluids.

To our knowledge, this study is the first to compare the baseline skin and gill mucus metabolic profiles in fish. The sum of glucogenic amino acids and sum of biogenic amine concentrations were significantly different in the two types of mucus. Thus, the concentrations of amino acids were higher in gill mucus relative to skin mucus, which might be attributed to the increased protein metabolism capacities and osmotic potential of gills [23]. Among the top 10 metabolites that differentiated gill and skin mucus, several amino acids such as alanine, glutamine and glutamic acid were more abundant in gill mucus as compared to skin mucus, which could be related to functions like osmoregulation and ammoniagenesis of gill tissue [23,24]. By using the japanese medaka (*Oryzias latipes*) as a model species, the important functions of the glutamic acid/glutamine cycle in controlling osmoregulation in gills has been reported [25]. The rapid accumulation of glutamic acid in gills was closely correlated both with the elevated levels of nitrogenous ammonia (NH_3_/NH_4_^+^) and urea after salinity challenge, contributing to the energetics of well-developed osmoregulatory abilities in euryhaline teleosts [25]. As an effective ammonia detoxification strategy reported in Chinese loach (*Paramisgurnus dabryanus*) followed by aerial exposure, glutamic acid was partially converted to alanine without releasing ammonia via transamination [26]. Considering that gills are a primary site of osmoregulation and excretion of nitrogenous waste, we assumed that metabolism of glutamic acid is higher in gill epithelium as compared to skin. One cannot exclude the possibility that increased glutamate levels in gill mucus may originate from both the gill tissue as well as from the serum. This assumption is supported by the observed correlation between biological fluids in the present study. In addition, the concentrations in biogenic amines were higher in gill mucus compared to skin mucus. This could indicate a higher capacity of the urea cycle and relatively increased activity of enzymes such as ornithine decarboxylase and S-adenylosyl methioninedecarboxylase that are involved in the generation of polyamines from ornithine [27]. Considering that the activities of those enzymes in skin and gill mucus of salmon are poorly investigated, additional studies should be initiated to explain the observed differences in the levels of these bioactive molecules that are derived from the urea cycle.

Outputs from the pathway analyses, based on the untargeted data, revealed several metabolites that could be affected by the anaesthetic benzocaine. These were alanine, isoleucine, leucine, glutamic acid, aspartate, proline and glucose. This is similar to another study in which sedation with clove oil affected the levels of glucose, alanine, proline, 4-hydroxyproline, leucine, isoleucine, and glutamic acid in the plasma of Chinook salmon (*Oncorhynchus tshawytscha*) [28]. Considering the correlation between biological fluids demonstrated in the present study, our findings support the use of non-invasive samples as mucus for the standardization of the sampling protocol minimizing the potential bias associated with the use of anesthetics.

## 4. Materials and Methods

### 4.1. Chemicals and Reagents

Benzocaine (BENZOAK, ACD Pharmaceuticals AS, Leknes, Norway), Optima LC−MS grade water, acetonitrile, isopropanol and methanol were provided by Fisher Scientific (Oslo, Norway). Ammonium carbonate was from Fluka (Steinheim, Germany), whereas phenyl isothiocyanate (PITC; ≥99%), was purchased from Sigma-Aldrich (St. Louis, MO, USA). The AbsoluteIDQ^®^ p400 HR Kit was provided by Biocrates Life Sciences AG (Innsbruck, Austria).

### 4.2. In Vivo Treatments and Fish Sampling

Atlantic salmon (*Salmo salar*; *n* = 10) weighing between 70–80 g were used in the current study. Fish were randomly divided into two groups (5 fish/each), and fish in one group were euthanized before sampling. For euthanization of fish, an overdose of benzocaine (200 mg/L) was used. Fish in the second group were killed using percussive stunning [29] and was considered as a control group. Skin and gill mucus samples were collected from both groups using absorption, according to Tartor et al. [30]. In brief, fish were placed on one side, and skin mucus was absorbed by covering the exposed surface with a piece of medical wipes (2.5 × 7 cm each; Kimberly-Clark, Irving, TX, USA). Mucus saturated wipes were gently removed and placed into the upper compartments of a 0.45 µm cellulose acetate Costar Spin-X centrifuge tube filters (Corning Inc., NY, USA). The Spin-X tubes were kept on ice until centrifugation (13,000× *g*, 4 °C, 10 min) to collect mucus filtrate. For gill mucus sampling, a single piece of medical wipe (0.5 × 1 cm) was placed for 5 s on the lateral surface of the gill filaments on each gill arch in both the left and right gill of each fish. The gill mucus-containing wipes were then processed as described above. Blood samples were collected from the caudal vein of the euthanized fish using vacutainer tubes (VACUETTE^®^, Greiner Bio-One, Frickenhausen, Germany) and were kept on ice prior to centrifugation (3000× *g*, 4 °C, 15 min) to separate plasma from blood cells. The mucus and plasma samples were kept at −80 °C until further processing and analyses.

### 4.3. Targeted and Untargeted Metabolomics

#### 4.3.1. Untargeted Metabolomics

At the day of the analysis, samples (*n* = 5, biological replicates) were thawed on ice and vortexed thoroughly for 15 s. Aliquots were transferred to the HPLC vials and placed randomly in the autosampler tray, which was kept at 8 °C.

#### 4.3.2. Targeted Metabolomics Using the AbsoluteIDQ^®^ p400 HR Kit

A targeted metabolomic analysis was performed by using the Biocrates kit to quantify up to 408 metabolites from eight metabolite classes including glycerophospholipids (196), glycerides (60), acylcarnitines (55), sphingolipids (40), amino acids (21), biogenic amines (21), cholesteryl esters (14) and sum hexoses (usually largely dominated by glucose) [31]. Considering the broad analyte coverage, Biocrates platform was chosen to improve the knowledge regarding to the metabolic composition of the epidermal mucus and correlate the metabolic profiles of epidermal mucus and plasma. The kit was prepared by following the detailed protocol provided by Biocrates. The total workflow was composed of six steps including addition of internal standards to the sample, derivatisation of amino acids using 5% phenyl isothiocyanate solution (PITC), filtration, extraction and dilution before instrumental analysis (see below).

The frozen (−80 °C) gill mucus, skin mucus and serum samples were placed on ice until completely thawed. The sample loading volume was 10 µL for both serum and mucus samples. The serum samples were additionally centrifuged at 2750× *g* for 5 min at 4 °C before loading on the plate. All samples were stored at −80 °C immediately after processing for the Absolute IDQ p400 HR kit. All measurements were carried out on three biological replicates.

Three quality control samples at three different levels (QC1, QC2 and QC3) were provided by Biocrates and used to ensure that quantification of the metabolites performed in this study was generally accurate and reproducible. QC2 samples were measured in replicates of 4 and used further for the normalization purpose.

#### 4.3.3. High-Resolution-Mass Spectrometry Analyses (HRMS)

All samples included in the present study were analysed using a Vanquish Horizon ultrahigh-performance liquid chromatography system (UHPLC) coupled to a Q-Exactive Fourier-transform high-resolution mass spectrometer (both Thermo Fisher Scientific, Bremen, Germany), equipped with a heated electrospray interface (HESI-II). The AbsoluteIDQ p400 HR kit allows simultaneous quantification of 408 metabolites by using two different instrumental approaches, i.e., UHPLC using a octadecylsilane column (ODS; Biocrates) and flow injection analysis (FIA), followed by HRMS analysis [31]. Amino acids and biogenic amines were separated on the ODS column using a water/acetonitrile (both containing 0.2% formic acid) gradient according to the manufacturer’s protocol. Total UHPLC analysis time was approximately 6 min per sample. All other substances including acylcarnitines, glycerophospholipids, sphingolipids, sum hexoses, cholesterol esters and glycerides were analysed using a shotgun approach by FIA–HRMS with a total analysis time of approximately 3.8 min per sample. The mobile phase was prepared according to the manufacture instruction diluting the FIA mobile phase buffer with LC−MS grade methanol. The source parameters used for LC–HRMS analysis were as follows: sheath gas flow rate 60, auxiliary gas flow rate 30, sweep gas flow rate 1, spray voltage 3 kV, capillary temperature 300 °C, S-lens RF level 60, and auxiliary gas heater temperature 550 °C. The source parameters used for FIA–HRMS were as follows: sheath gas flow rate 15, auxiliary gas flow rate 5, sweep gas flow rate 1, spray voltage 2.5 kV, capillary temperature 300 °C, S-lens RF level 60, and auxiliary gas heater temperature 120 °C. The data collection was performed in the full scan mode both for the LC–HRMS and FIA–HRMS analyses. Prior to the HRMS analyses, the instrument performance was confirmed by the system stability test completed according to the manufacture instructions.

For the untargeted analysis, a zwitterionic SeQuant ZIC-pHILIC column (Merck, Kenilworth, NJ, USA; 150 × 4.6 mm, 5 µm) was employed for hydrophilic interaction chromatography (HILIC) using a mobile phase consisting of 20 mM ammonium carbonate (pH 8.3; (A) and acetonitrile (B). The flow rate was set to 0.3 mL/min, and the column eluted using the following gradient: 80% B for 1 min followed by a decrease to 20% B over 29 min. After flushing the column with 8% B for 5 min, the mobile phase composition was returned to starting conditions and equilibrated for 9 min. The injector flushing solvent was 50% acetonitrile, the seal wash solvent consisted of 75% isopropanol, 25% water and 0.1% formic acid.

The mass spectrometer was run in positive and negative ion mode using fast polarity switching (i.e., alternating positive and negative ion scans), and scanned in the mass range *m*/*z* 58–870. The mass resolution was set to 70,000 at *m*/*z* 200. The spray voltage was 2.8 and 3.0 kV (positive and negative mode, respectively), the transfer capillary temperature was 280 °C, and the sheath and auxiliary gas flow rates were 35 and 10 units, respectively. Xcalibur software (Thermo Fisher Scientific, Waltham, MA, USA) was used for instrument control (version 2.3) and calculation of elemental compositions and mass errors (version 4.2).

### 4.4. Data Post-Processing

**Figure 6:**
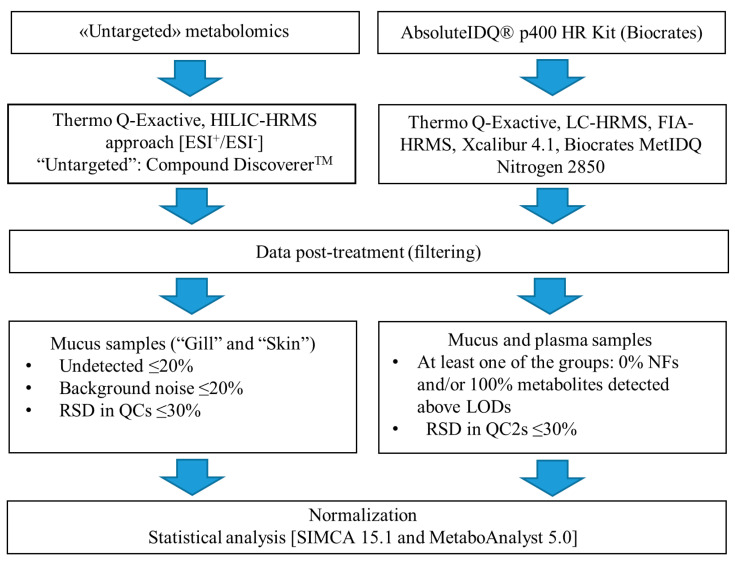
Flow chart overview of data collection and post-processing.

The data processing is divided into two parts. The targeted and untargeted metabolomics data were proceed separately as presented in Figure 6.

#### 4.4.1. Targeted Metabolomics: AbsoluteIDQ^®^ p400 HR Kit

The MetIDQ software (Biocrates, Life Science AG, Innsbruck, Austria) was used to process raw data prior to the statistical analyses. Considering the potential differences in metabolic signature, separate sets of data were prepared for mucus and plasma samples. As we were interested in all potential metabolites that might be present in skin and gill mucus samples, a considerable proportion of low abundance metabolites (<LOD values) was allowed. Furthermore, we retained only metabolites with 100% non-zero replicate measurements within at least one of the groups included in the present study, and/or metabolites that were detected in concentrations above the LOD for all individuals within at least one of the groups studied. Missing values were filled with one-third of the calculated LOD values.

Both for mucus and plasma samples, the raw data were normalized in order to control and correct for technical variability. This was achieved by using metabolite-specific correction factors that were calculated using the plates’ replicated QC2 samples (*n* = 4). For each metabolite, the median QC2 concentration (above LOD) was divided by the metabolite target value, given by the assay manufacturer and available in the MetIDQ database. Then, the sample concentrations (only for samples above LOD) were divided by the metabolite-specific correction factors. An additional filtration step was introduced into the workflow excluding metabolites with a relative standard deviation (RSD) of >30% among QCs. For mucus samples, the data were then normalized by median to reduce impact of differential dilution during the sampling procedure.

#### 4.4.2. Untargted Metabolomics

The Compound Discoverer 3.1 software was used to process untargeted raw data (Table S7). The workflow included, among others, spectra selection, retention time alignment, detection and grouping of unknown compounds, filling of missing values and compounds annotation, while the list of potential targets was generated and manually curated (Figure 6) before using MeatboAnalyst 5.0 and SIMCA 16.0 (Sartorius Stedim Biotech, Umeå, Sweden) for statistical analysis.

### 4.5. Statistical Analysis

The processed targeted and untargeted data were evaluated using univariate and multivariate statistical analyses. In our final workflow, the data was median-normalized, log-transformed and Pareto scaled. The univariate analyses were performed using MetaboAnalyst 5.0 [26] and included one-way ANOVA followed by *t*-test, fold change analysis and volcano plots. Volcano plot analysis included a false discovery rate (FDR) adjusted) *p*-value ≤ 0.1 and fold change cut-off of 2, respectively. MetaboAnalyst was used to generate heatmaps to visualise the top 10 discriminant metabolites selected by the *t*-test/ANOVA. Multivariate modelling of the Pareto-scaled data, including unsupervised principal component analysis (PCA) and supervised orthogonal partial least squares discriminant analysis (OPLS-DA), were used to assess the general distribution of the samples and identify potential outliers, and find metabolites with discriminating power, respectively.

## 5. Conclusions

Fish mucus has recently received significant interest as a non-invasive tissue for biochemical analyses. Mucus on accessible mucosal surfaces including skin and gills can be collected in a relatively simple and non-invasive manner, providing relevant information about the health status of the fish. Although it is well known that epidermal mucus contains several components associated with innate immunity, there is still a significant gap of knowledge regarding the potential of epidermal mucus to reflect the physiological responses of fish in general. Contrary to the proteomic profile associated with the immune mechanisms, the fish mucus metabolome is poorly investigated and there is a clear lack of data regarding to the functional interactions between biological fluids of fish. To our knowledge, for the first time the basic metabolic profiles of skin and gill mucus were compared with the metabolic profile of plasma by using quantitative metabolomics. A high level of correlation between plasma and mucus was demonstrated, opening new possibilities for non-invasive, quick and simple sampling protocols to collect information on fish health and welfare.

## Figures and Tables

**Table 1:** The number of metabolites with an intra-group variation RSD ≤ 30% from untargeted HILIC–HRMS.

Normalization Method	N (RSD ≤ 30%) ^1^				Median RSD (%) ^2^			
	Controls ^3^		Benzocain		Controls ^3^		Benzocaine	
	Gills	Skin	Gills	Skin	Gills	Skin	Gills	Skin
Non-normalized	719	724	811	909	32	33	30	24
Sum of peak areas	845	807	825	894	28	30	29	27
Protein content	358	170	72	868	40	48	66	28
Median	853	908	815	932	28	25	30	25
Median fold change	853	913	838	938	28	25	29	24

^1^ Number of metabolites with an intra-group RSD ≤ 30%; ^2^ Intra-group median RSD; ^3^ Fish killed by percussive stunning.

**Table 2:** The quality parameters of OPLS-DA models associated with normalization-by-median and median fold change (MFC) methods.

OPLS-DA ^1^	R2Y		Q2Y		CV-ANOVA [*p* Value]		Permutation
	Median	MFC	Median	MFC	Median	MFC	Median/MFC
Control_Skin vs. Benzocaine_Skin	0.927	0.929	0.833	0.834	0.0019	0.0019	Valid
Control_Gills vs. Benzocaine_Gills	0.95	0.948	0.757	0.756	0.0071	0.0071	Valid
Benzocaine_Skin vs. Benzocaine_Gills	0.906	0.905	0.874	0.873	0.0007	0.0007	Valid
Control_Skin vs. Control_Gills	0.927	0.935	0.833	0.863	0.0019	0.0010	Valid

^1^ All models were generated with one predictive component and zero orthogonal components.

**Table 3:** The major metabolic pathways that were expressed differently in skin and gill mucus or disturbed due to the exposure to benzocaine.

Groups Comparison	Metabolic Pathways	*p* Value	Significant Hits
Control_Skin vs. Benzocaine_Skin	Aminoacyl-tRNA biosynthesis	0.009	L-phenylalanine, L-alanine, L-lysine, L-isoleucine, L-aspartate, L-proline
Control_Gills vs. Benzocaine_Gills	Fructose and mannose metabolism	0.021	D-fructose, D-mannose, D-glyceraldehyde, 6-deoxy-L-galactose, D-glucose, 2-dehydro-3-deoxy-L-fuconate, D-lactic acid
	beta-Alanine metabolism	0.022	glycerone, D-lactate, D-glyceraldehyde, 3-hydroxypropanoate, β-alanine, L-aspartate, 3-aminopropanal, L-alanine
Benzocaine_Skin vs. Benzocaine_Gills	Alanine, aspartate and glutamate metabolism	0.006	N-acetyl-L-aspartate, L-aspartate, D-aspartate, L-alanine, L-glutamic acid, 4-aminobutanoate, L-glutamine, fumaric acid, β-citryl-L-glutamate
	Aminoacyl-tRNA biosynthesis	0.028	L-phenylalanine, L-glutamine, glycine, L-aspartate, L-alanine, L-isoleucine, L-leucine, L-threonine, L-proline, L-glutamic acid
	Pyrimidine metabolism	0.033	L-glutamine, thymine, (*R*)-3-ureidoisobutyrate, β-alanine, (*R*)-3-aminoisobutyrate
	Glutathione metabolism	0.033	glutathione, glycine, L-glutamic acid, 5-oxoproline, L-ornithine
	Selenocompound metabolism	0.041	L-alanine, β-alanine, sarcosine
Control_Skin vs. Control_Gills	β-Alanine metabolism	0.024	3-hydroxypropanoate, β-alanine, L-aspartate,3-ureidopropionate, dihydrouracil
	Aminoacyl-tRNA biosynthesis	0.039	L-asparagine, L-phenylalanine, glycine, L-aspartate, L-methionine, L-alanine, L-lysine, L-isoleucine, L-leucine, L-glutamic acid

**Table 4:** Number of detected metabolites, organized by compound class, and the most abundant metabolite per class in salmon gill, skin mucus and plasma samples detected by using the AbsoluteIDQ^®^ p400 HR kit.

	Total Number of Metabolites	Detected		Most Abundant		
		Mucus	Plasma	Gill	Skin	Plasma
Acylcarnitines [AC (X:Y)]	55	3	10	L-Carnitine		
Amino Acids [AA]	21	19	20	L-Glutamic acid	L-Valine	L-Valine
Biogenic Amines [BA]	21	5	10	Taurine		
Lysophosphatidylcholines[LPC (X:Y)]	24	-	12	ND	ND	LPC (22:6)
ConfirmedPhosphatidylcholines [PC (X:Y)]	172	8	54	PC (38:6)	PC (34:2)	PC (38:6)
Ceramides [Cer (X:Y)]	9	-	1	ND	ND	Cer (42:2)
Sphingomyelins [SM (X:Y)]	31	1	10	SM (42:3)	SM (42:3)	SM (42:2)
Sum hexoses [including glucose]	1	1	1	Sum hexoses		
Cholesteryl Esters [CE (X:Y)]	14	-	7	ND	ND	CE (22:6)
Diglycerides [DG (X:Y)]	18	-	10	ND	ND	DG (36:2)
Triglycerides [TG (X:Y)]	42	1	28	TG (52:7)	TG (52:7)	TG (56:7)

ND: Not detected.

## Data Availability

The authors were unable to find a valid data repository for the data used in this study. Please contact the corresponding author for raw data sharing.

## Supplementary Materials

The following are available online at https://www.mdpi.com/article/10.3390/metabo12010028/s1, Figure S1: Clustering skin and gill mucus samples represented as a dendrograms, Figure S2: Relative differences between gill and skin mucus metabolites from salmon euthanized using benzocaine or percussive stunning (“Control”), Absolute IDQp400 kit, Figure S3 Heatmap (*t*-test/ANOVA) exhibiting different concentration patterns of metabolites in skin and gill mucus, Absolute IDQp400 kit, Table S1: Total protein content in gill and skim mucus samples, Table S2: Metabolite concentrations in skin and gill mucus, Absolute IDQ p400 kit, Table S3: Plasma metabolites detected using the Absolute IDQ p400 kit; Table S4: The ratios of metabolites that significantly differentiated the skin and gill mucus samples; Table S5: Significantly different metabolites, gill vs. skin mucus, Volcano plot; Table S6: Significantly different metabolites, gill vs. skin mucus collected following benzocaine treatment; Table S7: Compound Discoverer 3.1 software key settings used for untargeted data processing.
