# Supplementary material for: Fish Skin and Gill Mucus: A Source of Metabolites for Non-Invasive Health Monitoring and Research
Source: Metabolites. 2021 Dec 31;12(1):28. doi: 10.3390/metabo12010028 (PMC8781917; doi:10.3390/metabo12010028)
Supplement: Supplementary file 1 [file metabolites-12-00028-s001.zip › metabolites-1501494-supplementary.pdf]

Supplementary Material

Fish Skin and Gill Mucus: A Source of Metabolites for Non-Invasive Health Monitoring and Research

Lada Ivanova *, Oscar D. Rangel-Huerta, Haitham Tartor, Mona C. Gjessing, Maria K. Dahle and Silvio Uhlig

Norwegian Veterinary Institute, P.O. Box 64, N-1431 Ås, Norway
** Correspondence: lada.ivanova@vetinst.no*

TABLE OF CONTENTS

Table S1. Total protein content in gill and skin mucus samples p.S2

Table S2. Metabolite concentrations in skin and gill mucus, Absolute IDQ p400 kit p. S3

Table S3. Plasma metabolites detected using the Absolute IDQ p400 kit p. S4–7

Table S4. The ratios of metabolites that significantly differentiated the skin and gill mucus samples. Metabolic significance is based on the information provided by Biocrates p. S7

Table S5. Significantly different metabolites, gill vs. skin mucus, Volcano plot p. S8

Table S6. Significantly different metabolites, gill vs. skin mucus collected following benzocaine treatment, Volcano plot p. S8

Table S7. Compound Discoverer 3.1 software key settings used for untargeted data processing p. S8–10

Figure S1. Clustering skin and gill mucus samples represented as a dendrograms (distance measure used is Euclidean and clustering algorithm is ward). The impact of normalization on the total variability of mucus samples p. S11

Figure S2. Relative differences between gill and skin mucus from salmon euthanized using benzocaine or percussive stunning (“Control”), Absolute IDQ p400 kit p. S12

Figure S3. Heatmap (t-test/ANOVA) exhibiting different concentration patterns of metabolites in skin and gill mucus collected following percussive stunning or b enzocaine treatment. The top-10 most differential metabolites are shown, Absolute IDQ p400 kit p. S12

Table S1. Total protein content (mg/ml) in the skin and gill mucus samples

Sample ID	Total protein, mg/ml
Benzocaine_Gills_Fish 1	3.6
Benzocaine_Gills_Fish 2	0.6
Benzocaine_Gills_Fish 3	1.6
Benzocaine_Gill_Fish 4	2.3
Benzocaine_Gills_Fish 5	1.5
Control_Gills_Fish 1	1.7
Control_Gills_Fish 2	1.6
Control_Gills_Fish 3	1.8
Control_Gills_Fish 4	0.9
Control_Gills_Fish 5	1.4
Benzocaine_Skin_Fish 1	0.5
Benzocaine_Skin_Fish 2	0.3
Benzocaine_Skin_Fish 3	0.4
Benzocaine_Skin_Fish 4	0.4
Benzocaine_Skin_Fish 5	0.6
Control_Skin_Fish 1	0.2
Control_Skin_Fish 2	0.5
Control_Skin_Fish 3	0.5
Control_Skin_Fish 4	0.5
Control_Skin_Fish 5	1.4

Table S2. Metabolites that were quantified in gill and skin mucus using the Absolute IDQp400 kit. The concentration levels are median values (μM) of three biological replicates.

			Treatment			
			Skin		*Gill*	
Compound		*Class*	*Control*	*Benzocaine*	*Control*	*Benzocaine*
Carnitine	AC(0:0)	Acylcarnitines	4.60	8.47	5.55	9.44
Acetylcarnitine	AC(2:0)	Acylcarnitines	2.29	2.03	3.03	2.55
Fumarylcarnitine	AC(4:1-DC)	Acylcarnitines	0.003	0.01	0.03	0.01
Alanine	Ala	Amino Acids	1.57	1.61	13.7	28.3
Arginine	Arg	Amino Acids	1.40	1.07	4.56	6.98
Aspartate	Asp	Amino Acids	6.52	5.32	7.59	32.6
Glutamine	Gln	Amino Acids	5.53	7.13	13.5	23.9
Glutamate	Glu	Amino Acids	6.01	6.02	39.7	78.1
Glycine	Gly	Amino Acids	7.03	6.90	21.0	29.7
Histidine	His	Amino Acids	1.19	1.34	2.18	2.49
Isoleucine	Ile	Amino Acids	2.83	0.94	7.60	8.23
Lysine	Lys	Amino Acids	NF	NF	4.49	8.44
Methionine	Met	Amino Acids	2.28	2.36	3.85	6.19
Ornithine	Orn	Amino Acids	0.94	1.00	0.90	0.98
Phenylalanine	Phe	Amino Acids	1.32	1.30	3.07	5.07
Proline	Pro	Amino Acids	2.02	2.26	5.33	7.66
Serine	Ser	Amino Acids	2.56	5.17	8.07	18.7
Threonine	Thr	Amino Acids	4.42	0.86	18.0	11.9
Tryptophan	Trp	Amino Acids	0.57	0.64	0.69	1.04
Tyrosine	Tyr	Amino Acids	0.85	0.94	1.79	3.26
Valine	Val	Amino Acids	8.51	5.98	15.4	17.3
Leucine + Isoleucine	xLeu	Amino Acids	5.01	2.30	21.6	24.0
Putrescine	Putrescine	Biogenic Amines	1.95	0.94	7.93	10.4
Sarcosine	Sarcosine	Biogenic Amines	NF	NF	1.18	1.57
Spermidine	Spermidine	Biogenic Amines	0.13	0.07	0.46	0.75
trans-4-Hydroxyproline	t4-OH-Pro	Biogenic Amines	0.27	0.89	0.89	3.91
Taurine	Taurine	Biogenic Amines	89.2	127	418	577
PC(34:2)	PC(34:2)	Phosphatidylcholines	0.14	0.003	0.13	0.12
PC(35:2)	PC(35:2)	Phosphatidylcholines	0.04	0.04	0.05	0.05
PC(36:4)	PC(36:4)	Phosphatidylcholines	0.04	NF	0.09	0.25
PC(36:5)	PC(36:5)	Phosphatidylcholines	0.06	0.001	0.16	0.17
PC(36:6)	PC(36:6)	Phosphatidylcholines	0.04	0.03	0.08	0.08
PC(38:6)	PC(38:6)	Phosphatidylcholines	0.04	0.03	0.90	0.79
PC(40:9)	PC(40:9)	Phosphatidylcholines	0.04	0.04	0.16	0.13
PC(41:5)	PC(41:5)	Phosphatidylcholines	0.04	0.05	0.05	0.05
SM(42:3)	SM(42:3)	Sphingomyelins	0.04	0.04	0.04	0.05
Hexoses (including glucose)	H1	Monosaccharides	204	121	664	480
TG(52:7)	TG(52:7)	Triglycerides	2.75	2.03	2.43	2.88

Table S3. Overview of salmon plasma metabolites and lipids using the AbsoluteIDQ® p400 kit. The concentration levels are given as median values (μM) of three biological replicates.

		Treatment	
		Plasma	
Compound	*Class*	*Control*	*Benzocaine*
AC(0:0)	Acylcarnitines	3.45	2.38
AC(2:0)	Acylcarnitines	2.79	1.12
AC(12:0)	Acylcarnitines	0.02	0.03
AC(12:1)	Acylcarnitines	0.01	0.02
AC(14:0)	Acylcarnitines	0.04	0.05
AC(16:0)	Acylcarnitines	0.04	0.02
AC(16:1)	Acylcarnitines	0.03	0.02
AC(18:0)	Acylcarnitines	0.04	0.02
AC(18:1)	Acylcarnitines	0.17	0.14
AC(18:2)	Acylcarnitines	0.03	0.05
Ala	Aminoacids	389	761
Arg	Aminoacids	84.7	116
Asp	Aminoacids	15.0	21.0
Cit	Aminoacids	2.61	12.8
Gln	Aminoacids	224	431
Glu	Aminoacids	110	100
Gly	Aminoacids	323	412
His	Aminoacids	64.9	65.1
Ile	Aminoacids	571	458
Lys	Aminoacids	337	211
Met	Aminoacids	72.4	137
Orn	Aminoacids	22.2	21.0
Phe	Aminoacids	88.3	114
Pro	Aminoacids	36.8	123
Ser	Aminoacids	76.9	197
Thr	Aminoacids	649	309
Trp	Aminoacids	20.8	18.1
Tyr	Aminoacids	41.3	58.2
Val	Aminoacids	1290	861
xLeu	Aminoacids	1054	870
ADMA	Biogenic amines	0.35	0.37
Creatinine	Biogenic amines	17.5	14.7
Met-SO	Biogenic amines	0.64	9.65
Putrescine	Biogenic amines	17.4	12.7
Sarcosine	Biogenic amines	1.27	1.63
SDMA	Biogenic amines	0.49	0.54
Spermidine	Biogenic amines	1.05	0.76
Spermine	Biogenic amines	0.32	0.35
t4-OH-Pro	Biogenic amines	24.9	83.0
Taurine	Biogenic amines	615	479
LPC(15:0)	Lysophosphatidylcholines	1.52	1.67

LPC(16:0)	Lysophosphatidylcholines	20.2	20.9
LPC(16:1)	Lysophosphatidylcholines	2.67	3.00
LPC(17:1)	Lysophosphatidylcholines	0.08	0.12
LPC(18:0)	Lysophosphatidylcholines	0.57	0.71
LPC(18:1)	Lysophosphatidylcholines	3.61	5.82
LPC(18:2)	Lysophosphatidylcholines	0.28	0.62
LPC(20:1)	Lysophosphatidylcholines	0.44	1.72
LPC(20:4)	Lysophosphatidylcholines	1.44	2.58
LPC(22:6)	Lysophosphatidylcholines	25.1	24.1
LPC(24:1)	Lysophosphatidylcholines	1.01	3.10
LPC-O(18:1)	Lysophosphatidylcholines	1.04	1.45
PC(25:0)	Phosphatidylcholines	0.03	0.03
PC(30:0)	Phosphatidylcholines	1.05	1.28
PC(30:1)	Phosphatidylcholines	3.93	4.50
PC(31:2)	Phosphatidylcholines	0.26	0.52
PC(32:1)	Phosphatidylcholines	11.0	15.3
PC(32:2)	Phosphatidylcholines	1.50	2.72
PC(32:3)	Phosphatidylcholines	0.39	2.11
PC(32:4)	Phosphatidylcholines	0.13	0.36
PC(33:1)	Phosphatidylcholines	4.49	6.58
PC(33:4)	Phosphatidylcholines	0.73	1.09
PC(34:1)	Phosphatidylcholines	82.3	105
PC(34:2)	Phosphatidylcholines	33.8	73.6
PC(34:3)	Phosphatidylcholines	8.22	20.4
PC(34:4)	Phosphatidylcholines	9.80	13.8
PC(34:5)	Phosphatidylcholines	8.20	22.2
PC(35:2)	Phosphatidylcholines	2.77	4.95
PC(35:3)	Phosphatidylcholines	0.23	0.55
PC(35:4)	Phosphatidylcholines	0.66	0.89
PC(35:5)	Phosphatidylcholines	5.99	11.5
PC(36:2)	Phosphatidylcholines	20.6	42.6
PC(36:3)	Phosphatidylcholines	15.8	51.4
PC(36:4)	Phosphatidylcholines	55.9	1.27
PC(36:5)	Phosphatidylcholines	23.0	49.1
PC(36:6)	Phosphatidylcholines	0.47	0.79
PC(37:1)	Phosphatidylcholines	0.04	0.11
PC(37:3)	Phosphatidylcholines	0.90	1.22
PC(37:4)	Phosphatidylcholines	0.77	1.74
PC(37:5)	Phosphatidylcholines	3.65	5.21
PC(37:6)	Phosphatidylcholines	8.96	13.97
PC(37:7)	Phosphatidylcholines	1.49	2.78
PC(38:4)	Phosphatidylcholines	0.03	30.5
PC(38:6)	Phosphatidylcholines	623	725
PC(39:4)	Phosphatidylcholines	0.94	1.86
PC(39:5)	Phosphatidylcholines	0.68	1.59
PC(40:2)	Phosphatidylcholines	1.57	1.61
PC(40:4)	Phosphatidylcholines	1.62	3.54
PC(40:9)	Phosphatidylcholines	3.12	0.96

PC(41:5)	Phosphatidylcholines	2.40	3.45
PC(41:8)	Phosphatidylcholines	0.29	0.31
PC(42:7)	Phosphatidylcholines	3.03	8.71
PC(43:6)	Phosphatidylcholines	0.64	0.81
PC-O(32:1)	Phosphatidylcholines	0.22	0.53
PC-O(33:2)	Phosphatidylcholines	0.36	0.87
PC-O(34:1)	Phosphatidylcholines	1.50	1.83
PC-O(34:2)	Phosphatidylcholines	1.36	1.83
PC-O(35:3)	Phosphatidylcholines	0.21	0.47
PC-O(36:2)	Phosphatidylcholines	2.02	2.31
PC-O(36:3)	Phosphatidylcholines	1.19	1.66
PC-O(36:4)	Phosphatidylcholines	2.39	2.28
PC-O(36:5)	Phosphatidylcholines	7.48	10.7
PC-O(36:6)	Phosphatidylcholines	0.90	1.36
PC-O(37:7)	Phosphatidylcholines	7.67	14.8
PC-O(38:5)	Phosphatidylcholines	0.04	5.96
PC-O(38:6)	Phosphatidylcholines	40.9	41.4
Cer(42:2)	Ceramides	3.93	5.31
SM(31:0)	Sphingomyelins	0.05	0.07
SM(32:1)	Sphingomyelins	4.45	6.98
SM(34:1)	Sphingomyelins	1.37	1.75
SM(38:2)	Sphingomyelins	6.02	18.7
SM(40:2)	Sphingomyelins	8.86	8.64
SM(41:1)	Sphingomyelins	0.75	1.60
SM(41:2)	Sphingomyelins	1.12	2.15
SM(42:2)	Sphingomyelins	57.2	82.6
SM(43:2)	Sphingomyelins	1.61	1.39
SM(44:2)	Sphingomyelins	0.87	0.54
H1	Monosaccharides	27110	6780
CE(16:0)	Cholesteryl Esters	61.5	120
CE(16:1)	Cholesteryl Esters	82.5	151
CE(17:1)	Cholesteryl Esters	4.49	5.96
CE(17:2)	Cholesteryl Esters	3.86	5.23
CE(18:2)	Cholesteryl Esters	49.4	107
CE(18:3)	Cholesteryl Esters	29.3	60.3
CE(22:6)	Cholesteryl Esters	3800	5119
DG(32:1)	Diglycerides	2.06	2.66
DG(34:1)	Diglycerides	4.23	8.38
DG(34:3)	Diglycerides	1.10	2.57
DG(36:2)	Diglycerides	4.46	8.47
DG(36:3)	Diglycerides	2.94	4.04
DG(36:4)	Diglycerides	1.53	1.82
DG(42:0)	Diglycerides	0.11	0.31
DG(44:3)	Diglycerides	0.08	0.25
DG-O(34:1)	Diglycerides	0.82	2.24
DG-O(36:4)	Diglycerides	0.18	0.50
TG(48:1)	Triglycerides	4.45	16.4
TG(48:2)	Triglycerides	3.39	15.4

TG(49:1)	Triglycerides	1.32	5.00
TG(49:2)	Triglycerides	0.97	2.33
TG(50:2)	Triglycerides	25.7	71.9
TG(50:3)	Triglycerides	15.8	46.5
TG(50:4)	Triglycerides	13.5	23.9
TG(51:2)	Triglycerides	4.34	14.0
TG(51:3)	Triglycerides	5.01	13.2
TG(51:4)	Triglycerides	2.56	4.93
TG(52:2)	Triglycerides	39.1	93.8
TG(52:3)	Triglycerides	40.3	105
TG(52:4)	Triglycerides	23.2	58.5
TG(52:5)	Triglycerides	13.8	30.6
TG(52:6)	Triglycerides	15.9	24.4
TG(52:7)	Triglycerides	5.58	9.10
TG(53:3)	Triglycerides	1.04	2.81
TG(53:5)	Triglycerides	2.59	4.73
TG(53:6)	Triglycerides	2.85	5.45
TG(54:3)	Triglycerides	69.1	186
TG(54:4)	Triglycerides	49.4	131
TG(54:5)	Triglycerides	44.4	86.4
TG(54:6)	Triglycerides	56.9	75.1
TG(55:7)	Triglycerides	8.70	9.17
TG(55:8)	Triglycerides	2.86	3.54
TG(56:6)	Triglycerides	0.20	10.7
TG(56:7)	Triglycerides	324	298
TG(56:8)	Triglycerides	22.7	20.4

Table S4. The ratios of metabolites that significantly differentiated the skin and gill mucus samples. Metabolic significance is based on the information provided by Biocrates.

Ratios and sums	*P*-value	Metabolic significance
Ratio of glutamate (Glu) to glutamine (Gln)	0.0334	Indicator of glutaminolysis rate and mitochondrial glutaminase activity
methionine (Met) to phenylalanine (Phe)	0.0169	An indicator linked to the folate metabolism
Sum of solely glucogenic amino acids (ΣGAAs)	0.0146	An indicator for gluconeogenic activity and endogenous glucose production
Ratio of ornithine (Orn) to arginine (Arg)	0.0190	An indicator of arginase activity and linked to urea cycle
Ratio of sum of polyamines (putrescine, spermidine and spermine) to ornithine (Orn)	0.0081	Polyamine turnover
Ratio of putrescine to ornithine (Orn)	0.0082	Indicator of ornithine decarboxylase activity
Sum of biogenic amines (ΣBAs)	0.0056	Feed associated indicator

Table S5. Metabolites that were significantly different in skin and gill mucus, inferred using the AbsoluteIDQ® p400 kit. Metabolites were selected from a Volcano plot under the conditions of fold change ≥2 and false discovery rate-adjusted P-value threshold ≤ 0.1. The P values were transformed by −log10.

	FC	log2(FC)	p.ajusted	-log(10)
Ala	11.3	3.5	0.0399	1.4
AC(4:1-DC)	3.88	2.0	0.0399	1.4
Orn	0.33	-1.6	0.0399	1.4

Table S6. Metabolites that were significantly different in skin and gill mucus collected following benzocaine treatment. Univariate analysis was performed by using by Volcano plot based on a fold change ≥2 and false discovery rate-adjusted P-value threshold ≤ 0.1. The P-values are transformed by −log10.

	FC	log2(FC)	p.ajusted	-log(10)
Ala	9.76	3.29	0.0449	1.35
Sarcosine	4.75	2.25	0.0449	1.35
PC(41:5)	0.27	-1.91	0.0449	1.35
PC(35:2)	0.29	-1.79	0.0449	1.35
AC(0:0)	0.34	-1.54	0.0449	1.35
Glu	2.64	1.40	0.0449	1.35
AC(2:0)	0.42	-1.25	0.0449	1.35
PC(36:5)	31.6	4.98	0.0946	1.02

Table S7. Compound Discoverer 3.1 software key parameters used during the untargeted data processing.

**Compound Discoverer 3.1 worflow**	
**Select spectra**	
1. General Settings:	
Precursor Selection:	Use MS(n - 1) Precursor
Provide Profile Spectra	Automatic
2. Spectrum Properties Filter:	
Lower RT Limit [min]	0.2
Upper RT Limit [min]	30
First Scan	0
Last Scan	0
Ignore Specified Scans	not Specified
Lowest Charge State	0
Highest Charge State	0
Min. Precursor Mass [Da]	58
Max. Precursor Mass [Da]	870
Total Intensity Threshold	0
Minimum Peak Count	1
3. Scan Event Filters:	
Mass Analyzer	not Specified
MS Order	Any
Activation Type	not Specified
Min. Collision Energy	0

Max. Collision Energy	1000
Scan Type	is Full
Polarity Mode	Any
4. Peak Filters:	
S/N Threshold [FT-only]	1.5
5. Replacements for Unrecognized Properties:	
Unrecognized Charge Replacements	1
Unrecognized Mass Analyzer Replacements	ITMS
Unrecognized MS Order Replacements	MS2
Unrecognized Activation Type Replacements	CID
Unrecognized Polarity Replacements	+
Unrecognized MS Resolution@200 Replacements	60000
Unrecognized MSn Resolution@200 Replacements	30000

<u>Retention time alignment</u>

1. General Settings:	
Alignment Model	Adaptive curve
Alignment Fallback	Use Linear Model
Maximum Shift [min]	0.3
Shift Reference File	True
Mass Tolerance [ppm]	5
Remove Outlier	True

<u>Detect Compounds</u>

1. General Settings:		
Mass Tolerance [ppm]	5	
Intensity Tolerance [%]	30	
S/N Threshold	5	
Min. Peak Intensity	500000	
Ions	<u>Positive</u>	<u>Negative</u>
	$[2M+H]^{+1}$	$[M-2H]^{-2}$
	$[2M+K]^{+1}$	$[M-2H+K]^{-1}$
	$[2M+Na]^{+1}$	$[M-H]^{-1}$
	$[M+2H]^{+2}$	$[2M-H]^{-1}$
	$[M+H]^{+1}$	$[M+FA-H]^{-1}$
	$[M+H-H_2O]^{+1}$	$[M+H+HAc]^{-1}$
	$[M+H-NH_3]^{+1}$	$[M-H-H_2O]^{-1}$
	$[M+K]^{+1}$	
	$[M+Na]^{+1}$	
	$[M+NH_4]^{+1}$	
Base Ions	$[M+H]^{+1}$	$[M-H]^{-1}$
Min. Element Counts	C, H	
Max. Element Counts	C90H190Br3Cl4K2N10Na2O15P3S5	
2. Peak Detection:		
Filter Peaks	True	
Max. Peak Width [min]	0.8	
Remove Singlets	True	
Min. # Scans per Peak	5	

Min. # Isotopes	1	
**Group Compounds**		
1. Compound Consolidation:		
Mass Tolerance [ppm]	5	
RT Tolerance [min]	0.2	
2. Fragment Data Selection:		
Preferred Ions	$[M+H]^{+1}$	$[M-H]^{-1}$
**Fill Gaps**		
1. General Settings:		
Mass Tolerance [ppm]	5	
S/N Threshold	1.5	
Use Real Peak Detection	True	
**Normalize Areas**		
1. QC-based Area Correction:		
Regression Model	Linear	
Min. QC Coverage [%]	50	
Max. QC Area RSD [%]	30	
Max. # Files Between QC Files	15	
2. Area Normalization:		
Normalization Type	None	
Exclude Blanks	True	
3. Scaling Factor:		
Study Factor Name	not Specified	
**Mark Background Compounds**		
1. General Settings:		
Max. Sample/Blank	5	
Max. Blank/Sample	0	
Hide Background	True	

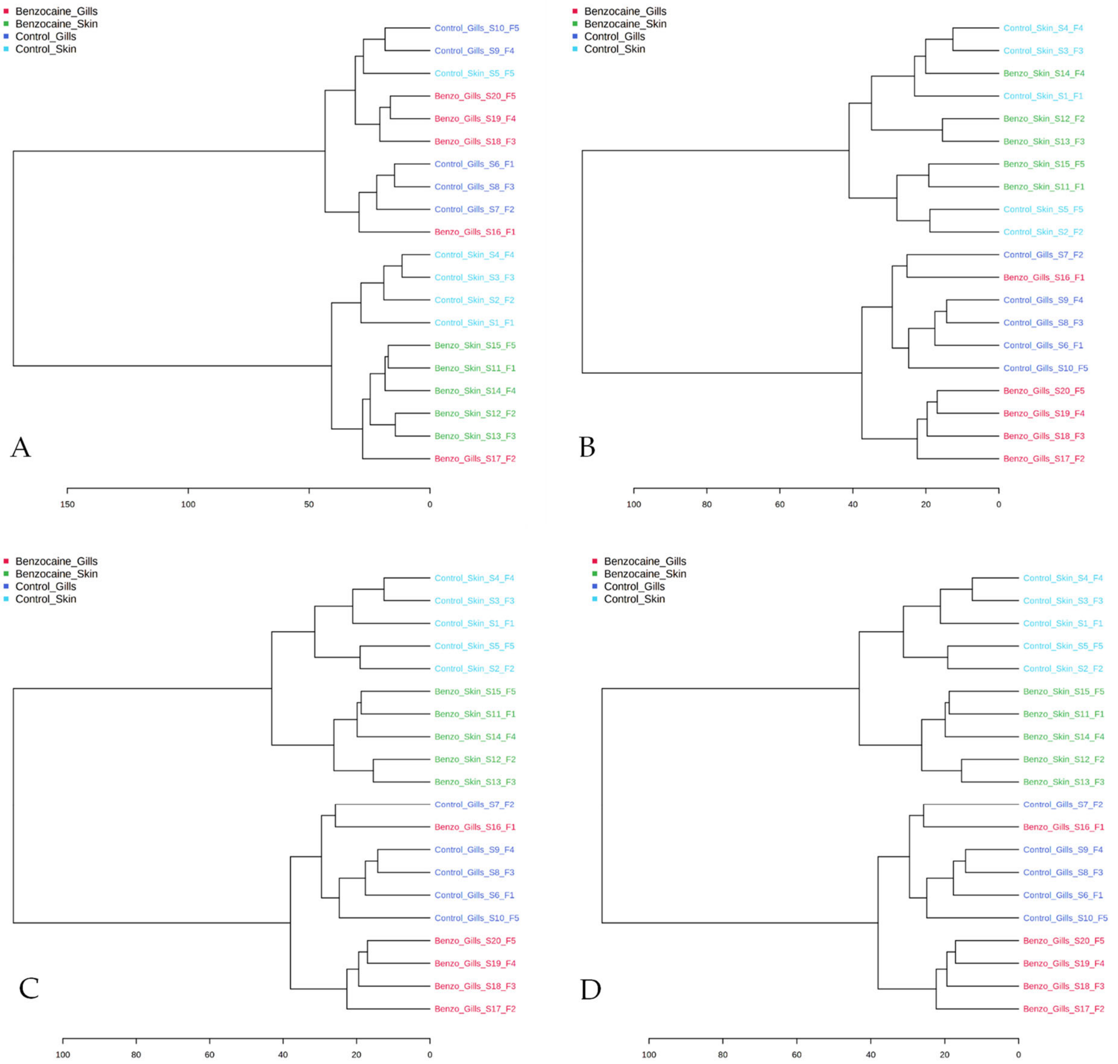

Figure S1. Clustering skin and gill mucus samples represented as a dendrograms (distance measure used is Euclidean and clustering algorithm is ward). The impact of normalization on the total variability of mucus samples collected from salmon euthanized using benzocaine or percussive stunning ("Control"). The data were normalized by (A) by plasma, (B) by sum, (C) by median and (D) by median fold change.

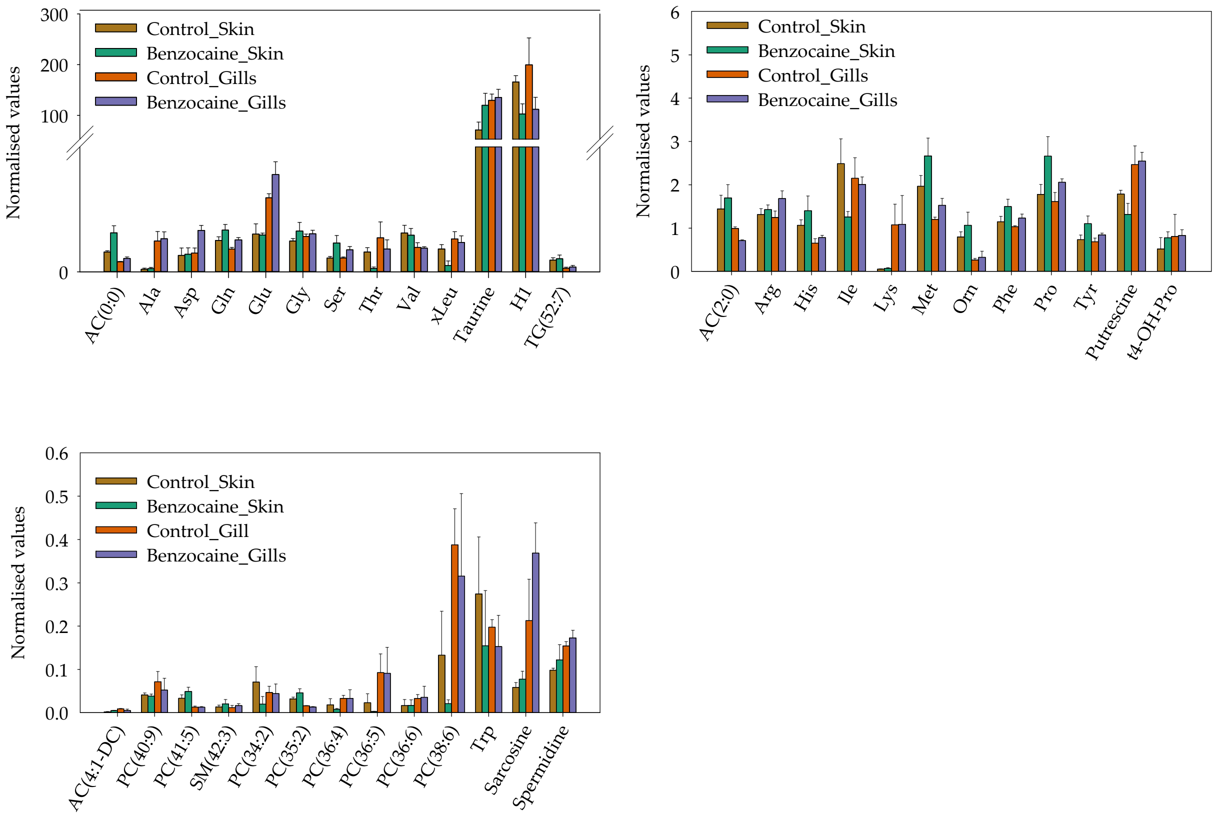

Figure S2. Relative differences between gill and skin mucus from salmon euthanized using benzocaine or percussive stunning ("Control"). The metabolite levels (µM) detected with the AbsoluteIDQ® p400 HR kit were normalised by median method. The data are presented as the mean, error bars are the standard error.

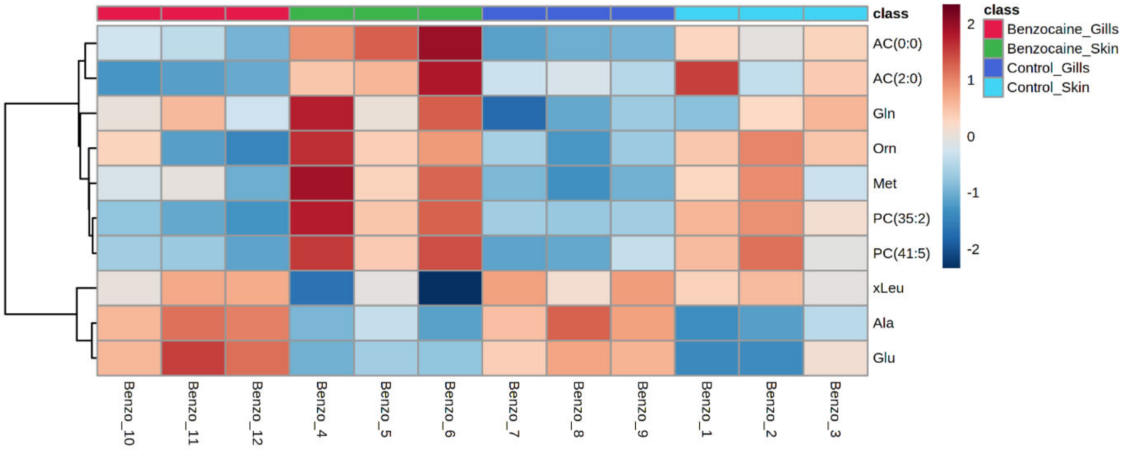

Fig. S3. Heatmap exhibiting the different distribution patterns of metabolites, obtained using the AbsoluteIDQ® p400 HR kit, in skin and gill mucus collected following percussive stunning or benzocaine treatment. The top-10 metabolites based on ANOVA/t-test are included.
